# Perinatal obesity primes the hepatic metabolic stress response in the offspring across life span

**DOI:** 10.1038/s41598-025-90082-4

**Published:** 2025-02-21

**Authors:** Sarah K Stegmann, Christina Vohlen, Nam Gyu Im, Jana Niehues, Jaco Selle, Ruth Janoschek, Celien Kuiper-Makris, Sonja Lang, Münevver Demir, Hans-Michael Steffen, Alexander Quaas, Jan-Wilm Lackmann, Dirk Nierhoff, Christoph Neumann-Haefelin, Jörg Dötsch, Miguel A Alejandre Alcazar, Philipp Kasper

**Affiliations:** 1 Department of Gastroenterology and Hepatology, Faculty of Medicine and University Hospital Cologne, University of Cologne, Cologne, Germany; 2 Department of Pediatrics and Adolescent Medicine, Faculty of Medicine and University Hospital Cologne, University of Cologne, Cologne, Germany; 3 Department of Pediatric and Adolescent Medicine, Translational Experimental Pediatrics, Experimental Pulmonology, Faculty of Medicine and University Hospital Cologne, University of Cologne, Cologne, Germany; 4 Institute for Lung Health (ILH), University of Giessen and Marburg Lung Center (UGMLC), Member of the German Center for Lung Research (DZL), Gießen, Germany; 5 Department of Hepatology and Gastroenterology, Campus Virchow-Klinikum (CVK) and Campus Charité Mitte (CCM), Charité University Medicine Berlin, Berlin, Germany; 6 Department of Pathology, Faculty of Medicine and University Hospital Cologne, University of Cologne, Cologne, Germany; 7 Cologne Excellence Cluster On Cellular Stress Responses in Aging-Associated Diseases (CECAD), Faculty of Mathematics and Natural Sciences, University of Cologne, Cologne, Germany; 8 Cologne Excellence Cluster on Cellular Stress Responses in Aging-Associated Diseases (CECAD) and Center for Molecular Medicine Cologne (CMMC), Faculty of Medicine and University Hospital Cologne, University of Cologne, Cologne, Germany

**Keywords:** Perinatal obesity, Oxidative stress, Hepatokines, Liver metabolism, Developmental programming, Non-alcoholic fatty liver disease, Risk factors, Paediatric research

## Abstract

Perinatal obesity is associated with an increased risk of metabolic diseases and hepatic dysfunction in offspring. However, the underlying mechanisms of this metabolic programming remain incompletely understood. This study aimed to elucidate the influence of maternal obesity and early life exposure to high-fat diet on offspring liver phenotype, hepatokine profile, and key components of hepatic metabolism. To this end, we employed a murine high-fat diet-induced perinatal obesity model, investigating the offspring in early life and late adulthood. After exposure to perinatal obesity, the offspring showed a significantly increased body weight in early life with no histological signs of steatosis, but a dysregulated hepatokine profile. Proteomic profiling, followed by molecular analyses, revealed a decreased lipogenesis and increased fatty acid oxidation, suggesting a protective mechanism against the development of steatosis. These changes were accompanied by increased markers of lipid peroxidation and DNA damage, indicating increased oxidative stress. Concomitantly, the antioxidative enzymes catalase and superoxide dismutase 2 were significantly reduced and oxidative phosphorylation was impaired, implying an altered oxidative stress response. While changes in oxidative stress level were only detected in early life, the lipid metabolism was altered across life span. This metabolic programming could determine the resilience and susceptibility to chronic liver disease later in life.

## Introduction

Obesity is a global epidemic with considerably increasing prevalence in recent years^1^. This growing health burden is associated with an increasing incidence of obesity among women of reproductive age, including pregnant women^2,3^. Perinatal obesity is not only associated with an increased health risk for mothers^3,4^, but also causes numerous adverse consequences in offspring^5,6^. According to the developmental origins of health and disease (DOHaD) hypothesis, it is assumed that an adverse metabolic environment in utero and during early life influences the offspring’s susceptibility to metabolic diseases later in life, a phenomenon known as metabolic programming^7,8^. Epidemiological studies have shown that maternal overweight is linked to an increased risk for obesity, type 2 diabetes, impaired liver metabolism, and the development of metabolic dysfunction-associated steatotic liver disease (MASLD) in offspring^9–13^. However, the underlying mechanisms remain incompletely understood.

Given its key regulatory role in energy homeostasis, lipid-, and glucose metabolism, an altered liver metabolism not only affects the liver itself, but also mediates adverse systemic effects and disrupts the function of other organ systems. Hepatokines are liver secreted proteins which act in an auto-, para-, and endocrine manner and are known to orchestrate this interorgan-crosstalk. While it is well known that hepatokines are differentially expressed in patients with metabolic dysfunction-associated liver diseases, their expression and relevance in the context of an adverse in utero and early life metabolic environment remain unclear^14,15^.

Oxidative stress is considered a central player in chronic metabolic diseases^16,17^. Previous studies have shown that patients with metabolic diseases exhibit a pronounced imbalance between pro-oxidants and anti-oxidants, coupled with increased production of oxidizing molecules. This imbalance ultimately results in an accumulation of oxidative free radicals and reactive oxygen species (ROS)^18^. Significantly increased ROS levels disrupt cellular proteins, leading to various forms of cellular dysfunction. These disruptions include alterations in energy metabolism and cell signaling, decreased biological activity, immune activation, and inflammation^17^.

Several studies demonstrated an interplay between oxidative stress and metabolic derangements in patients suffering from MASLD^19–23^. However, the extent to which offspring liver oxidative metabolism is altered following maternal obesity and early life exposure to a high-caloric diet has not been fully understood. Furthermore, information on the interplay between hepatokine expression, hepatic metabolism and oxidative stress system is still lacking.

Therefore, this study aimed at investigating the short- and long-term effects of a high caloric intake during pregnancy and early life on the hepatic stress response analyzing metabolic and oxidative stress parameters in the offspring of a high-fat diet-induced perinatal obesity murine model.

## Results

### A perinatal high-fat metabolic environment leads to an altered phenotype in offspring

**Fig. 1:**
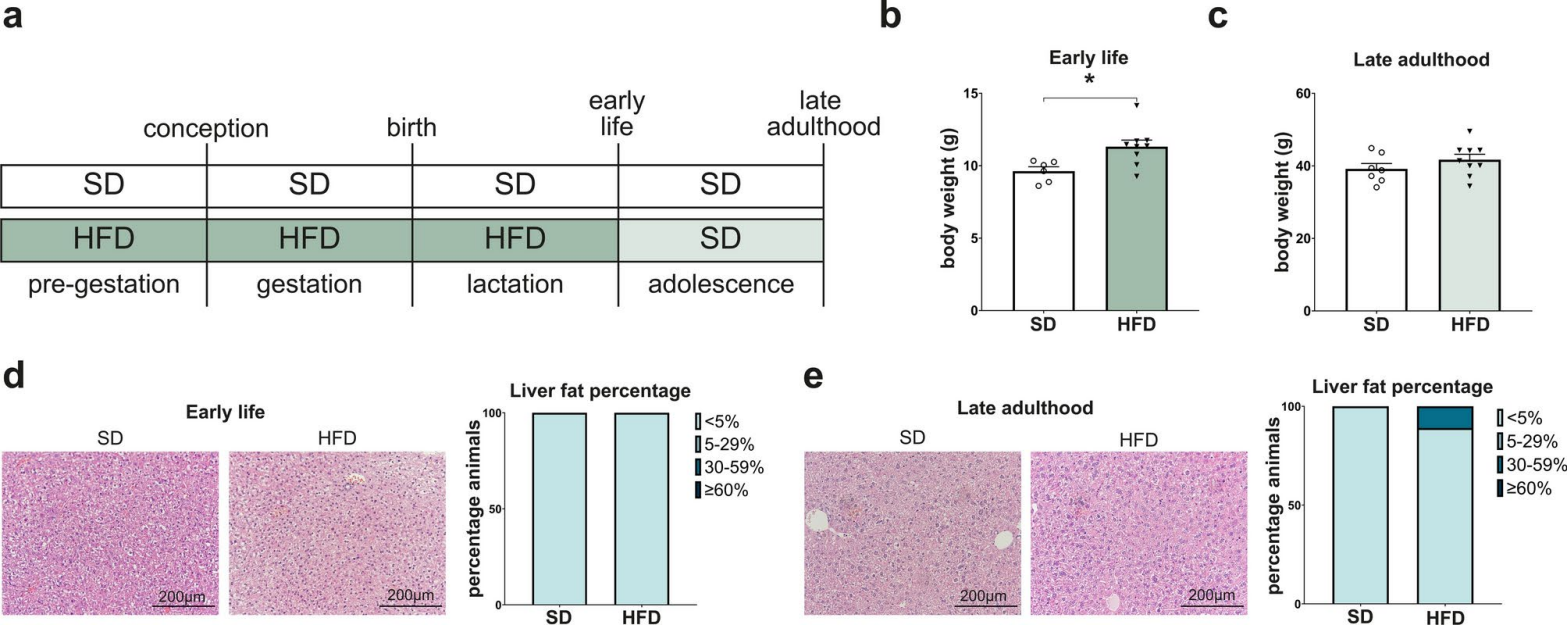
High-fat diet during pregnancy and early life leads to an obese phenotype in early life without signs of steatosis. (**a**) Scheme of the animal model in the present study: the perinatal obesity group received a high-fat diet (HFD) pre-gestationally, during pregnancy, and lactation. Offspring were weaned at postnatal day (P) 21 and either sacrificed or kept on standard diet (SD) until late adulthood (around 1.5 years). The control group was fed with SD. (**b**) Offspring of perinatal obesity display an increased bodyweight (n = 9) compared to the control group (n = 6). (**c**) Weight differences between the HFD group (n = 9) and control group (n = 7) diminish in late adulthood. (**d**) H&E histological analysis reveals no signs of steatosis in early life (SD n = 7, HFD n = 11). (**e**) H&E staining and analysis confirms no significant steatosis in late adulthood (SD n = 7, HFD n = 9). Data are presented as mean ± standard error of the mean (SEM). T-Test and Mann–Whitney-U-test were performed for statistical analysis of two groups and Fisher’s exact test and chi-square test were used for contingency tables. *p* < 0.05 was considered statistically significant and marked as *.

To investigate both the short- and long-term effects of a high-caloric metabolic environment during pregnancy and lactation on offspring liver health, we started feeding a high-fat diet (HFD) to virgin females 7 weeks prior to mating, resulting in an obese phenotype and impaired glucose tolerance in the dams as previously shown^24^. The offspring were then analyzed at two key time points: early life (after weaning, at postnatal day 21 (P21)) and late adulthood (approximately 1.5 years of age) (Fig. 1a). At the first timepoint of investigation (P21), the offspring showed a significantly increased body weight compared to the control group (*p* = 0.015, Fig. 1b). Despite a documented obese phenotype with increased visceral fat^25^, they exhibited hepatic fat accumulation in less than 5% of the hepatocytes in the histological analysis, therefore not fulfilling the characteristics of a significant steatosis (Fig. 1d).

After the offspring were weaned and fed standard diet (SD) until late adulthood, the body weight equalized to the control group (Fig. 1c). Histological analysis of the liver tissue samples also showed no significant increase in steatosis at 1.5 years of age (Fig. 1e). Altogether, these results demonstrate that perinatal obesity leads to an early life obese phenotype in offspring. This phenotype was not accompanied by hepatic steatosis at either time point, despite the perinatal oversupply of fatty acids.

### Perinatal obesity alters the hepatokine expression profile during early life

**Fig. 2:**
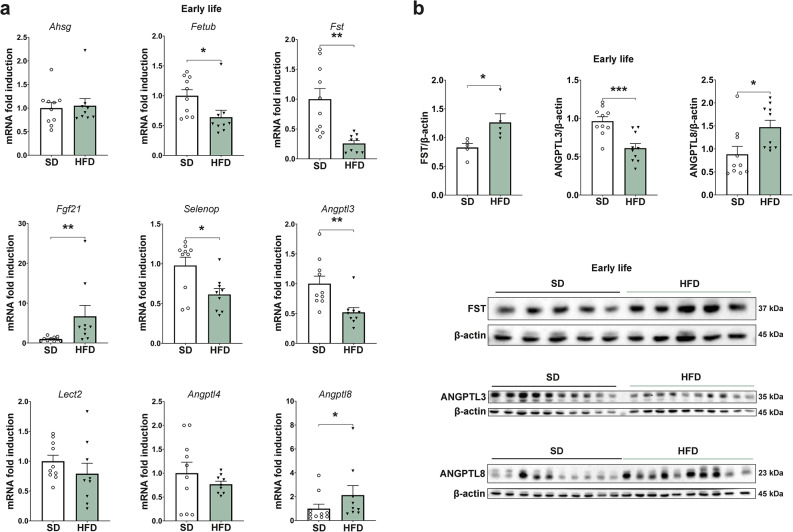
Perinatal obesity induces an altered hepatokine profile in early life. (**a**) Quantitative real-time PCR reveals a genetical regulation of common hepatokines in early life in the offspring of obese dams (n = 9, SD n = 10). (**b**) Protein levels of Follistatin (FST, n = 5 per group), Angiopoietin like 3 (ANGPTL3, n = 10 per group), and Angiopoietin like 8 (ANGPTL8, n = 10 per group) are significantly different in offspring of perinatal obesity analyzed by western blot in early life. Cropped images of representative blots of the protein and the respective loading control are shown below the graphs. Uncropped images of original blots are provided in Supplementary Fig. S5. Data are presented as mean ± SEM. T-test and Mann–Whitney U-test were performed with a statistical significance defined as *p* < 0.05 and labelled as *.

Since hepatokines play an important regulatory role in liver metabolism and are a main contributor to interorgan crosstalk^14,15^, we next assessed whether their expression is altered by an obese in utero and early-life environment. Here, we identified a differentially regulated hepatokine profile in early life. While the mRNA expression of Fetuin B (*Fetub*), Follistatin (*Fst*), Selenoprotein P (*Selenop*), and Angiopoietin like 3 (*Angptl3*) was significantly decreased, Fibroblast growth factor 21 (*Fgf21*) and Angiopoietin like 8 (*Angptl8*) were increased (Fig. 2a). The expression of Fetuin A (*Ahsg*), Leukocyte cell-derived chemotaxin 2 (*Lect2*), and Angiopoietin like 4 (*Angptl4*) was not affected (Fig. 2a). Upon examining protein levels, Follistatin was increased after perinatal obesity (1.27 ± 0.15 vs. 0.83 ± 0.07, *p* = 0.029, Fig. 2b left and top). Both angiopoietin-like proteins that were modified on an mRNA level, also showed a differential regulation on protein level, with ANGPTL3 being downregulated (0.61 ± 0.06 vs. 0.96 ± 0.058, *p* = 0.0005, Fig. 2b middle and middle) and ANGPTL8 being upregulated (1.47 ± 0.15 vs. 0.88 ± 0.17, *p* = 0.019, Fig. 2b right and bottom). To evaluate the extent to which changes of the hepatokine profile persist, the expression status was subsequently analyzed later in life after about 1.5 years. At that time point, only *Angptl3* mRNA expression was still significantly downregulated (Supplementary Fig. S1a). Follistatin, ANGPTL3, and ANGPTL8 protein expression was not differentially regulated (Supplementary Fig. S1b,c).

Previous work from our group has shown that female offspring, which were maintained under the same experimental conditions as males, do not exhibit increased body weight following perinatal obesity^25^. To determine sex-specific differences in liver metabolism, we measured the hepatokine expression in female offspring in early life. Fgf21 mRNA level was significantly increased (*p* = 0.0001), while other hepatokines were not differentially expressed (Supplementary Fig. S2). In summary, these data demonstrate that a perinatal HFD-environment induces sex-specific alterations in offspring hepatokine profile. These changes are evident in early life but do not persist after dietary changes.

### Proteomic profiling reveals differential regulation of pathways related to hepatic stress response including the lipid metabolism

**Fig. 3:**
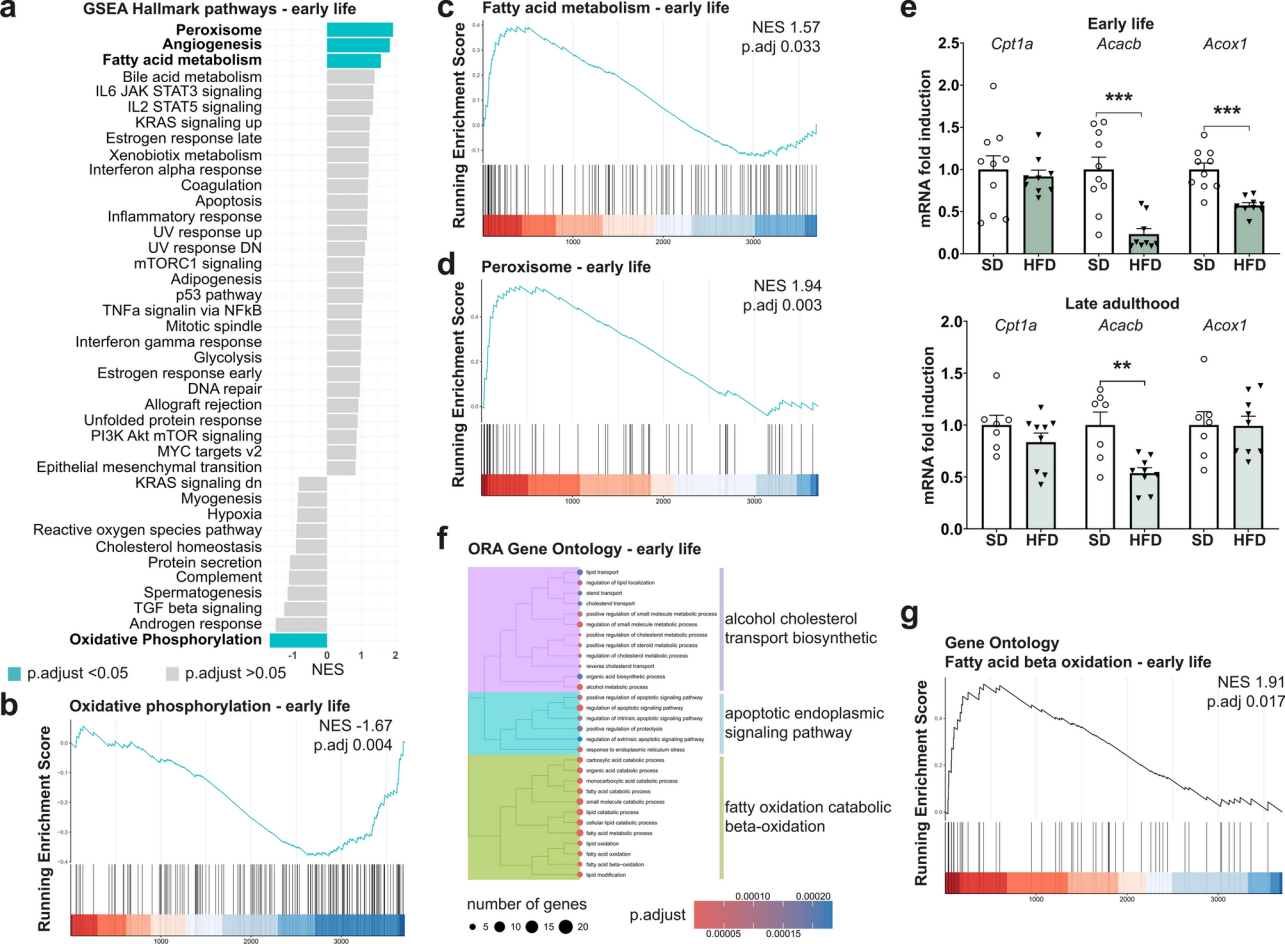
Offspring of obese dams display an adapted hepatic stress response with an increased fatty acid oxidation. (**a**) Hallmark pathway gene set enrichment analysis (GSEA) of proteomics data obtained in early life (n = 5 per group). ‘Peroxisome’, ‘Angiogenesis’, ‘Fatty acid metabolism’ and ‘Oxidative phosphorylation’ pathways are significantly altered in offspring exposed to perinatal obesity (NES—normalized enrichment score). (**b**) The GSEA plot shows a downregulation of ‘Oxidative phosphorylation’ in the offspring. (**c**) The ‘Fatty acid metabolism’ gene set is upregulated in early life. (**d**) The GSEA plot of ‘peroxisome’ shows an increased NES in the offspring of obese dams. (**e**) Quantitative real-time PCR in early life and late adulthood of key regulators of fatty acid oxidation (FAO) display an altered FAO metabolism that persists into late adulthood (early life: SD n = 10, HFD n = 9; late adulthood: SD n = 7, HFD n = 9). (**f**) Over-representation analysis (ORA) of significantly upregulated proteins using the ‘Gene Ontology’ (GO) gene sets. ‘Fatty oxidation catabolic/ beta-oxidation’ is one of the most differentially regulated processes in offspring of perinatal obesity in early life. (**g**) The GSEA plot of the GO pathway ‘Fatty acid beta oxidation’ demonstrates an enriched gene set in offspring of perinatal obesity in early life. Data are presented as mean ± SEM. For proteomics analysis, GSEA and ORA were performed and *p*.adjust < 0.05 was considered statistically significant. For statistical analysis of PCR-results, T-test or Mann–Whitney U-test were applied and statistical significance was defined as p < 0.05 and marked as *.

To analyze the influence of both an in utero and early life obesogenic diet and altered hepatokine profile on liver metabolism, we performed proteomic profiling during early life. We acquired a protein profile consisting of proteins that were present in all replicates of at least one condition, which allowed us to infer the corresponding genes and perform a gene set enrichment analysis (GSEA). Using the hallmark gene sets, we found the terms ‘peroxisome’, ‘angiogenesis’, ‘fatty acid metabolism’ and ‘oxidative phosphorylation’ to be significantly changed (Fig. 3a–d). ‘Fatty acid metabolism’ was upregulated (Fig. 3c), indicating an adaptive response of the lipid metabolism to an obese high-fat environment. As fatty acid beta-oxidation—a main part of fatty acid metabolism—takes place in peroxisomes, and the ‘peroxisome’ gene set was elevated in the GSEA, accordingly (Fig. 3d), we then performed PCR analysis on genes coding for key regulatory enzymes of fatty acid beta oxidation. This included Carnitine palmitoyltransferase 1a (*Cpt1a*), a rate-limiting enzyme, and Acetyl-CoA carboxylase beta (*Acacb*)—which produces Malonyl-CoA, an inhibitor of CPT1^19^. While *Cpt1a* was not differentially expressed on an mRNA level at either time point, *Acacb* was significantly decreased in early life at P21 (*p* = 0.0002), a change which persisted into late adulthood (*p* = 0.0022). *Acox1*, the gene coding for Acyl-CoA oxidase 1, a marker of peroxisomal fatty acid oxidation^19^, was significantly downregulated at the early time point but not in later (*p* = 0.0001, Fig. 3e). Additional over-representation analysis of upregulated proteins using the gene ontology (GO) gene sets and hierarchical clustering of enriched terms identified fatty acid beta oxidation as one of the main upregulated metabolic processes in the offspring as well as ‘alcohol cholesterol transport biosynthetic’ and ‘apoptotic endoplasmic signaling pathway’ (Fig. 3f). Moreover, GSEA based on the GO gene sets revealed 83 significantly different expressed pathways in the offspring (Supplementary Fig. S3) with ‘fatty acid beta oxidation’ being upregulated (Fig. 3g). Altogether, these data suggest an altered hepatic lipid metabolism with signs of upregulated fatty acid beta oxidation.

**Fig. 4:**
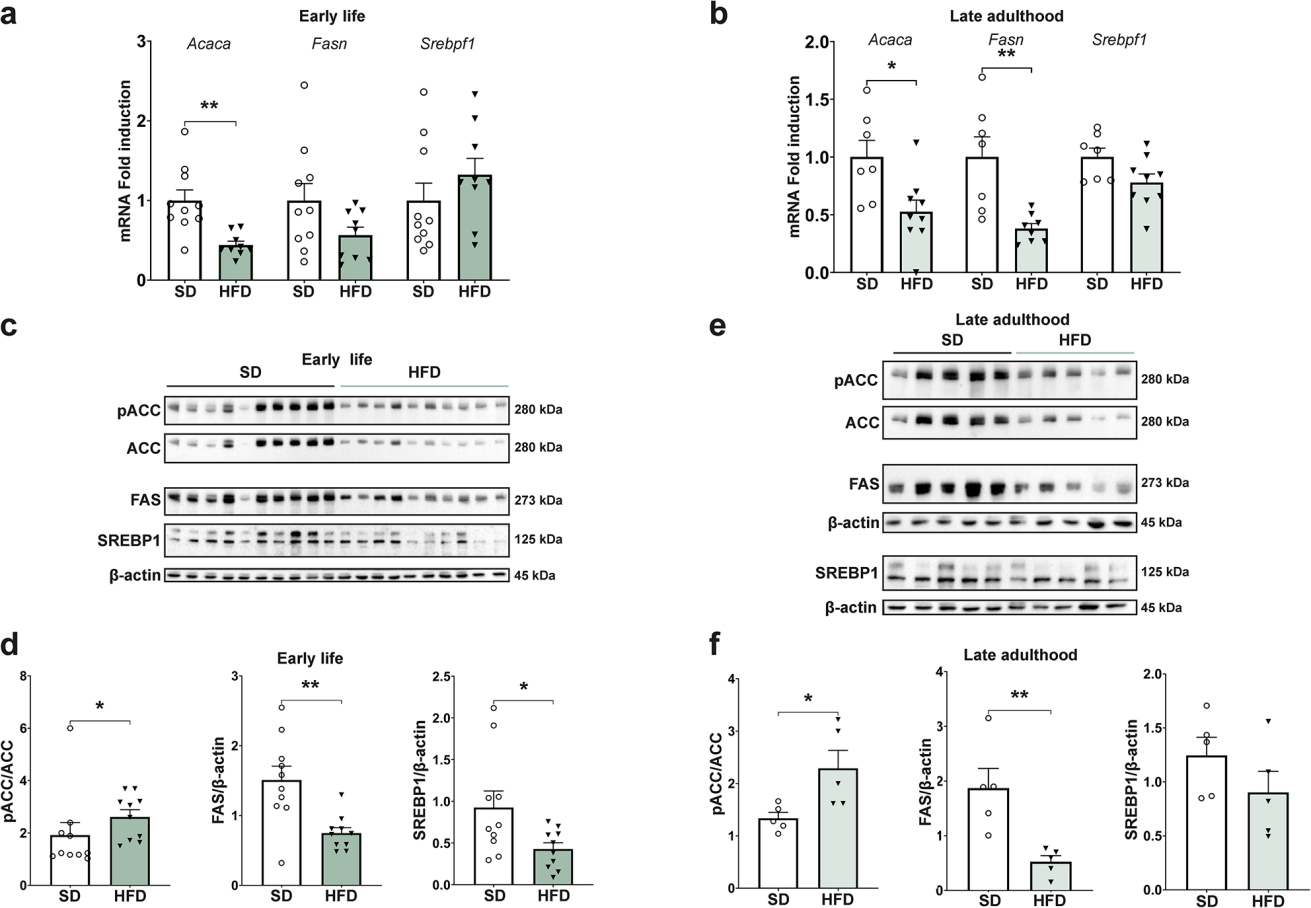
Perinatal obesity causes reduced lipogenesis in offspring that prevails into late adulthood. (**a**, **b**) Key regulators of lipogenesis are differentially expressed in early life (SD n = 10, HFD n = 9) and in late adulthood (SD n = 7, HFD n = 9) and analyzed by quantitative real-time PCR. (**c**, **d**) Representative western blots and statistical analysis of protein samples in early life display a downregulated lipogenesis in offspring of perinatal obesity (pACC—phosphorylated Acetyl-CoA carboxylase, ACC—Acetyl-CoA carboxylase, FAS—fatty acid synthase, SREBP1—sterol-regulatory element-binding protein 1; n = 10 per group). Cropped images of representative western blots of pACC/ACC as well as FAS and SREBP1 with the respective loading control are shown. Uncropped images of original blots are provided in Supplementary Fig. S5. (**e**, **f**) Reduction of lipogenesis prolongs into late adulthood in offspring of perinatal obesity analyzed by western blot (n = 5 per group). The displayed images show representative blots of pACC/ACC and FAS and SREBP1 each with their respective loading control. Uncropped images of original blots are provided in Supplementary Fig. S5. Data are presented as mean ± SEM. T-Test and Mann–Whitney U-Test were performed for statistical analysis and *p* < 0.05 was considered statistically significant and labelled as *.

Subsequently, we investigated hepatic lipogenesis in the offspring of obese dams. In early life, *Acaca,* which encodes for Acetyl-CoA Carboxylase alpha^19^, the key regulatory enzyme of lipogenesis^19^, was downregulated on an mRNA level (*p* = 0.0014), which lasted into late adulthood (*p* = 0.0147). mRNA expression of *Fasn* (Fatty acid synthase) was also significantly decreased in late adulthood (*p* = 0.0028), while no change in the expression of *Srebpf1*—a transcription factor activating lipogenesis—was detected (Fig. 4a,b)^19^. On a protein level, there was a significant increase in the phosphorylation of Acetyl-CoA Carboxylase (ACC), indicating an inactivation of the enzyme, at both time points (2.62 ± 0.27 vs. 1.92 ± 0.48, *p* = 0.0355 and 2.29 ± 0.34 vs. 1.34 ± 0.11, *p* = 0.03). Moreover, FAS was significantly reduced in early life and late adulthood (0.89 ± 0.09 vs. 1.54 ± 0.19, *p* = 0.0071 and 0.52 ± 0.11 vs. 1.87 ± 0.36, *p* = 0.0073) and Sterol-regulatory element-binding protein 1 (SREBP1) was downregulated in early life (0.43 ± 0.08 vs. 0.92 ± 0.20, *p* = 0.0308, Fig. 4c–f). These data indicate that the offspring exhibit an adapted lipid metabolism with signs of an increased fatty acid oxidation and decreased lipogenesis, which persists into late adulthood despite a dietary change to SD at weaning.

Similar alterations in lipid metabolism were detectable in female offspring. Acacb, an inhibitor of ACC, was downregulated in early life, as well as Acox 1 (Supplementary Fig. S4a)^19^. Key regulatory enzymes of lipogenesis were also reduced on mRNA level (Supplementary Fig. S4b). Thus, while there were sex-specific differences in hepatokine levels detectable in early life, the lipid metabolism response to the high-caloric environment during pregnancy and early life was similar between both sexes.

### Perinatal obesity alters the oxidative stress response in offspring during early life

**Fig. 5:**
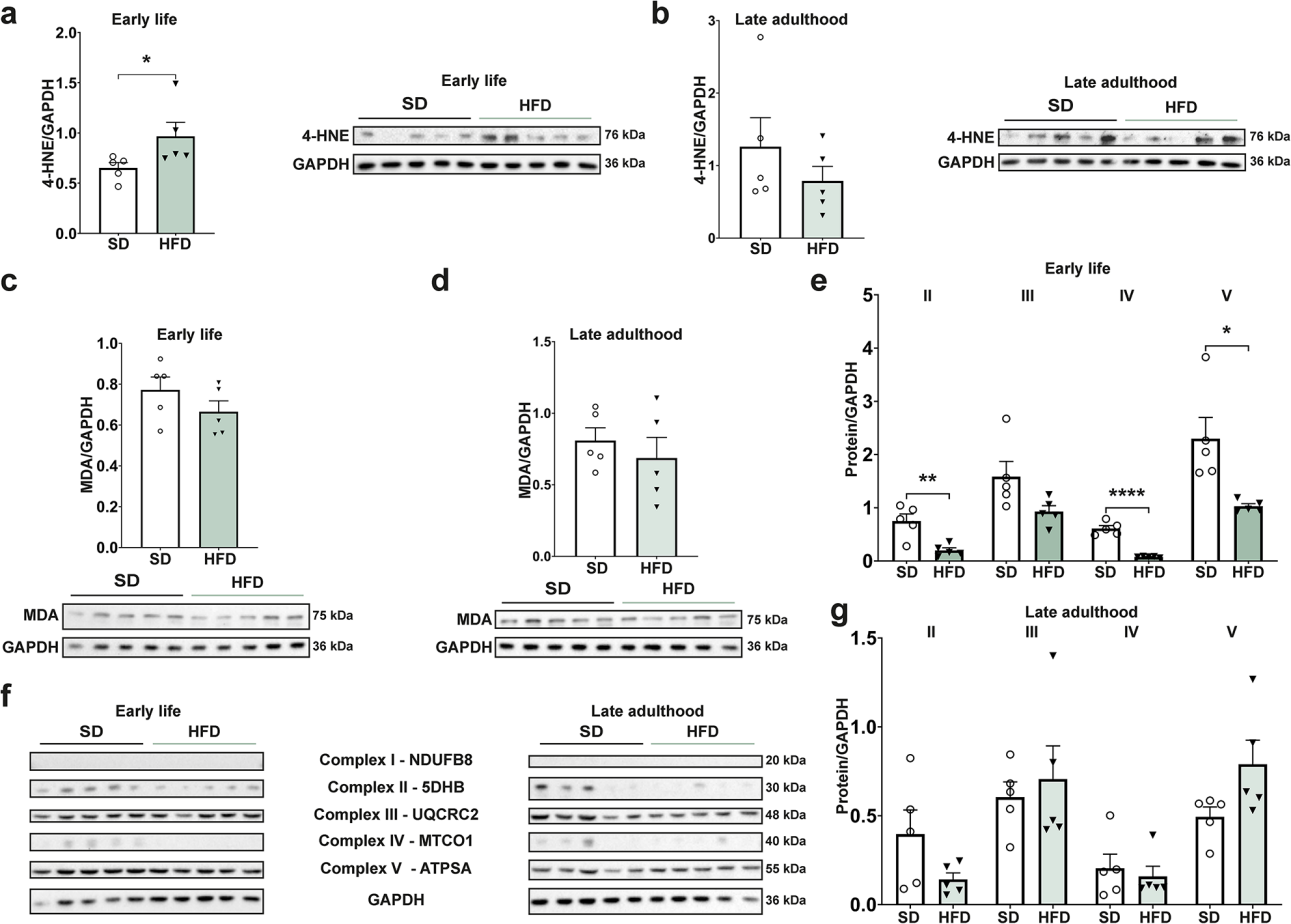
Offspring exposed to perinatal obesity display an increased oxidative stress level in early life which normalizes in late adulthood. (**a**, **b**) Western blot analysis of 4-Hydroxynonenal (4-HNE) as a marker of lipid peroxidation shows an increased oxidative stress level in early life that diminishes until late adulthood. Cropped images are shown with their respective loading control. (**c**, **d**) Malondialdehyde (MDA) as another marker of lipid peroxidation shows no alterations in early life or late adulthood. (**e**–**g**) Western blot analysis of complex I-V of oxidative phosphorylation. Analysis of complex II-V confirms a decreased oxidative phosphorylation in early life that normalizes in late adulthood. Complex I did not show up in all western blots. Representative blots are displayed with their respective loading control. The cropped images in (**f**) are shown at different exposure times for demonstration purposes. Uncropped images of original blots at the same exposure time are provided in Supplementary Fig. S5 (n = 5 for all groups). Data are presented as mean ± SEM. T-Test and Mann–Whitney-U-test were used and statistical significance was considered as *p* < 0.05 and marked as *.

**Fig. 6:**
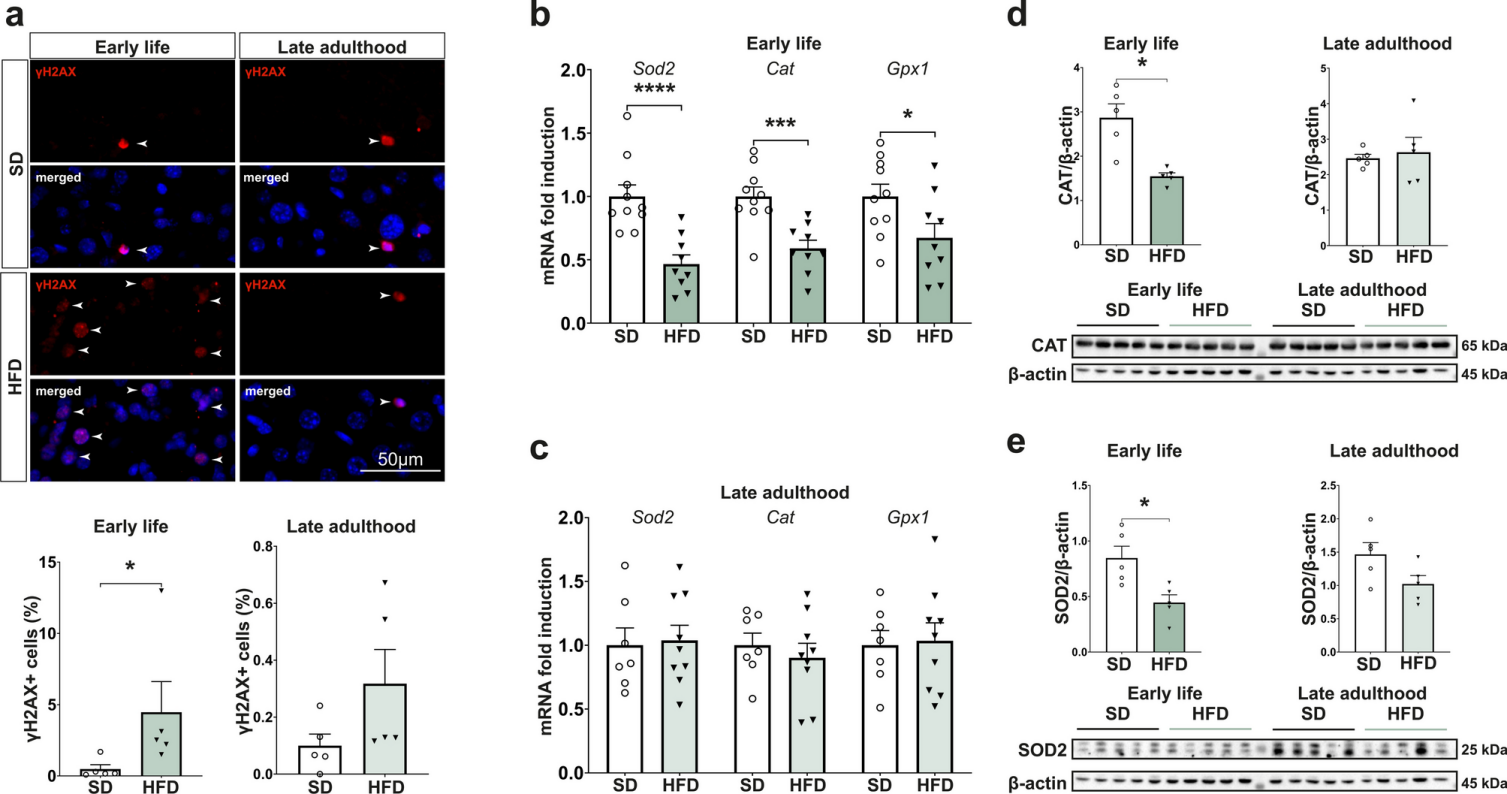
Perinatal obesity leads to an altered oxidative stress response in early life. (**a**) Immunofluorescence staining of liver sections reveals a significant increase of γ-H2A histone family member X (γH2AX) in early life but not in late adulthood. Representative images of each group are displayed and the quantification of γH2AX positive cells as a percentage of total cells was analyzed. γH2AX positive cells are indicated by an arrowhead. (**b**, **c**) mRNA levels of antioxidative enzymes are reduced in early life (SD n = 10, HFD n = 9) but equalize in late adulthood (SD n = 7, HFD n = 9). (**d**) Western Blot analysis reveals a significant decrease of catalase (Cat) in early life after perinatal obesity (n = 5 per group). Uncropped images of the representative blots are displayed in Supplementary Fig. S5. (**e**) Superoxidate dismutase 2 (Sod2) is reduced in early life but not in late adulthood on protein level (n = 5 per group). Representative images of Sod2 and the respective loading control are shown. Uncropped images are presented in Supplementary Fig. S5. Data are presented as mean ± SEM. T-Test and Mann–Whitney-U-test were used and statistical significance was defined as *p* < 0.05 and marked as *.

Since lipid metabolism and oxidative stress are closely linked^19^ and since chronic metabolic diseases are, in part, caused by an imbalance of pro-oxidants and anti-oxidants^17,18,22^, we next investigated markers of oxidative stress in the liver. On mRNA level, no changes in Integrin alpha M (*Itgam*), Tissue inhibitor of metalloproteinase 1 (*Timp1*) and Toll- like receptor 4 (*Tlr4*), known markers of oxidative stress in liver disease, were detectable neither at P21 nor in late adulthood (Supplementary Fig. S4e,f)^26^. To further asses oxidative stress levels, we next analyzed 4-Hydroxynonenal (4-HNE) and Malondialdehyde (MDA)—lipid peroxidation products formed by the oxidation of polyunsaturated fatty acids^22,27^. 4-HNE was significantly increased in early life (*p* = 0.0159, Fig. 5a), whereas no changes in MDA levels were detected (Fig. 5c)^22,27^. In late adulthood, both markers were not differentially expressed (Fig. 5b,d). As the GSEA of the hepatic proteomics data revealed a downregulation of oxidative phosphorylation (Fig. 3b), we next analyzed the different complexes of this system by western blot to further elucidate specific alterations of the electron transport chain reaction caused by the adverse metabolic environment. In early life, complex II, IV and V were significantly decreased compared to the control group (*p* = 0.0048, *p* < 0.0001 and *p* = 0.0134, Fig. 5e,f), but these changes did not persist into late adulthood (Fig. 5f,g). Complex I was not visibly expressed in any of the animal groups at different time points (Fig. 5c). Since increased oxidative stress can also lead to DNA damage^17^, we next assessed DNA double-strand breaks by measuring γ-H2A histone family member X (γH2AX), a sensitive marker of oxidative DNA damage repair^28^. γH2AX was significantly increased in early life in immunofluorescence staining (*p* = 0.0159), but these alterations diminished until late adulthood (Fig. 6a). Focusing on the antioxidative system, we found that the expression of genes encoding for superoxide dismutase 2 (*Sod2*), catalase (*Cat*), and glutathione peroxidase 1 (*Gpx1*) was reduced in early life (Fig. 6b,c). On a protein level, CAT and SOD2 were decreased at P21 but not in late adulthood (Fig. 6d,e). These results were, in part, reflected in the female population. At P21, *Itgam* was significantly increased, while *Timp1* and *Tlr4* were not altered (Supplementary Fig. S4c). *Cat* was significantly downregulated, but no changes in levels of *Sod2* and *Gpx1* could be detected (Supplementary Fig. S4d). Consequently, a maternal and early life HFD-environment leads to an increased oxidative stress level in the offspring that is accompanied by an altered oxidative stress response; however, these changes dissolve until late adulthood.

## Discussion

Based on observations from our previous studies that offspring of HFD-fed dams show an obese body composition throughout childhood and adolescence^24,25^, we investigated the effect of an in utero and early life adverse metabolic environment on the hepatic stress response in offspring throughout their lives. To this end, we performed an analysis of the expression profile of hepatokines as well as a global proteome analysis of the liver. In early life, we identified a pronounced, sex-specific dysregulation of the hepatokine profile as well as a significantly downregulated lipogenesis, and an increased expression of central nodal points of fatty acid beta oxidation. These alterations were accompanied by an increased oxidative stress level as well as an impaired oxidative phosphorylation and antioxidative regulatory machinery. While the observed changes of the hepatokine profile and oxidative metabolism were primarily present during critical windows of development early in life, the changes in lipid metabolism prolonged into late adulthood.

Maternal obesity increases the risk for several chronic metabolic diseases that evolve later in life for offspring, including obesity, type 2 diabetes and steatotic liver disease^11–13^. These metabolic changes often occur simultaneously, mediated by an interorgan crosstalk. Central mediators of this metabolic crosstalk are hepatokines, which exert pleiotropic effects on both lipid and glucose metabolism and are thought to be one of the key contributors to the development of metabolic liver diseases^15,29,30^. In the present study, the gene and protein expression of several important hepatokines was differentially regulated in the liver of the offspring of HFD-fed dams at P21, notably amongst them Fgf21, FST, ANGPTL3 and ANGPTL8. Moeckli et al. previously showed that mRNA levels of Fgf21 were significantly upregulated in male and female offspring’s livers of obese mothers at postnatal week 40^31^, consistent with our data at P21 and indicating a persistent change of expression even after weaning. In late adulthood after around 1.5 years, however, the differences diminish. The expression of ANGPTL3, ANGPTL4, and ANGPTL8 in the placenta of women with gestational diabetes has previously been shown to be positively correlated with birth weight of the offspring^32^. Offspring which are born small-for-gestational-age display the highest ANGPTL8 expression in venous cord blood^33^ and high levels of circulating ANGPTL8 in pregnant women are associated with an increased risk of gestational diabetes mellitus^34^. In the present study, offspring of obese dams also revealed an increased ANGPTL8 expression in early life, hinting at a HFD-associated placental dysfunction, which has also been associated with the development of an altered hepatic metabolism^35,36^. A high Follistatin expression has previously been detected in patients with MASLD and in vitro data suggests that a higher level of Follistatin is associated with a decreased lipogenesis^37^. Therefore, the increased Follistatin abundance in offspring of HFD-fed dams might contribute to the reduced lipogenesis observed in these mice. Additional studies are required to further explore this potential regulatory mechanism, to elucidate the pathways influenced by the observed changes in the hepatokine profile, and to understand the sex-specific differences detected in this study.

To date, previous work investigating the effects of perinatal obesity on hepatic metabolism in offspring are heterogeneous. While some studies have shown that offspring of obese dams exhibit signs of steatotic liver disease later in life, this phenotype and the cause remains inconsistent with either an increase in lipogenesis or a decreased fatty acid oxidation, being the ruling hypothesis^38–41^. In the present study, no significant histological indicators of hepatic lipid accumulation were evident. Analyses of lipid metabolism indicated that offspring of both sexes adapted their lipid metabolism to the increased nutrient supply via upregulation of fatty acid oxidation regulators and downregulation of lipogenesis. Our findings, which partially diverge from previous studies, might be attributed to a different diet and time points of analysis. While we used a high-fat diet containing 60% of metabolizable energy from fat, others have previously used diets with lower rates of fat^39,40,42,43^. At the same time, our diet only contained 24% of metabolizable energy derived from carbohydrates with 7% from sucrose. Thus, the caloric increase in our diet mostly derived from fat but not sugar. Recently, Janoschek et al.^44^ showed that the specific composition of a high-caloric diet is crucial for the development of a hepatic phenotype in offspring of obese dams. For example, they report steatosis only in diets with higher contents of both fat and sugar, but not in diets with high fat alone^44^. High sugar intake was also previously linked to increased hepatic lipogenesis in children^45,46^. Consistently, since we found decreased lipogenesis in our model of high-fat diet, these data show that the increase in fat alone leads to an altered hepatic metabolism but significant steatosis is only detectable if the intake in sugar is also increased. Further comparative studies are needed to elucidate whether an increase in sugar alone or the combination of sugar and fat are necessary to develop a steatotic phenotype in the offspring.

Since a perturbed metabolic milieu is associated with an altered oxidative system and chronic metabolic diseases are known to cause and influence oxidative stress^17,18^, we set out to determine to which extent the hepatic oxidative system is altered in the offspring exposed to a high-caloric intake during pregnancy and early life. Upregulated fatty acid oxidation, as shown in our data, is typically associated with an increase in ROS^19,47^. The generation of ROS eventually exceeds the capacity of the antioxidative defense system in the pathogenesis of metabolic-dysfunction associated steatotic liver disease^19^ and leads to mitochondrial dysfunction, as well as impairment of oxidative phosphorylation and lipid peroxidation, which promotes the development of the phenotype^47,48^.

In animal models, perinatal obesity and subsequent steatosis in offspring is linked to an increase in oxidative stress. McCurdy et al. demonstrated that nonhuman primate fetal livers of HFD-fed dams display elevated rates of the oxidative stress markers 8-hydroxy-deoxyguanisine and 4-HNE in utero (early in the third trimester)^42^. At 15 weeks of age, offspring of perinatal obesity present decreased activity of the electron transport chain in a murine model^39^. Moreover, Miranda et al. showed decreased activity of the antioxidative enzymes GPx, SOD and CAT along with an increase of oxidative stress markers in adult rat offspring (180 days old) after perinatal obesity^49^. In contrast to these studies, we did not only investigate one time point in prenatal or adult life, but rather demonstrated data for both adulthood and early life, a critical window of developmental programming. Here, an in utero and early life obesogenic environment resulted in increased oxidative stress levels, determined by an elevated lipid peroxidation and increased DNA damage, which was reflected by an upregulated DNA damage response. These alterations were accompanied by a decreased oxidative phosphorylation with downregulated complex II, IV and V, a mitochondrial dysregulation commonly seen in metabolic liver diseases^22^. Several antioxidative enzymes reducing ROS were impaired, further contributing to an increased oxidative stress level. Interestingly, the observed changes in the oxidative system were not evident after being fed a SD during adult life.

The following limitations must be considered when interpreting the present results: first, since the dams were fed an HFD during lactation and the offspring were not separated from their mothers until P21, they most likely also consumed the high-fat food a few days before weaning. Thus, this study does not investigate the influence of maternal obesity on the offspring alone but rather demonstrates the consequences of maternal obesity combined with early life exposure to a high-caloric metabolic environment. Further studies with an additional experimental group fed a SD after birth are needed in order to clearly differentiate the impact that each of these environmental conditions has on the liver phenotype seen in this study. Besides, as two representative time points were analyzed, the interpretation of dynamic metabolic changes is limited. Lastly, experiments with a second hit model such as exposure to HFD in adulthood are essential to further test the hypothesis of altered resilience in the offspring later in life. Nevertheless, as comprehensive analyses of the liver metabolism at early time points of human life remain difficult to obtain, this study provides deeper insights into regulatory mechanisms influenced by perinatal obesity.

In conclusion, the present work demonstrates that perinatal obesity reprograms molecular mechanisms underlying the hepatic metabolic stress response across life span. This metabolic hepatic programming could determine the resilience and susceptibility of the liver to metabolic as well as oxidative stressors, and ultimately to chronic liver disease later in life.

## Methods

### Animal model

All procedures were carried out under the Department of Pediatric and Adolescent Medicine of the University Hospital Cologne following the rules of the ‘Landesamt für Natur, Umwelt und Verbraucherschutz’ (LANUV), North Rhine-Westphalia, Germany and approved by the quoted authority (animal welfare application protocol number: 2012.A424; 2018.A230). The study complies with the Animals in Research: Reporting In Vivo Experiments (ARRIVE) guidelines^50^.

Animal procedures were performed as previously described^51^. Briefly, C57BL/6N mice were kept in the animal facility of the Department of Pharmacology, University Hospital Cologne, under temperature- und humidity-controlled 12-h dark/light cycle with libitum access to their respective chow and water. Young virgin female mice were fed with a high-fat diet (HFD, Altromin, Lage, Germany, catalog no. C1057, composed of 60% metabolizable energy (MetE) from fat, 16% MetE from protein and 24% of MetE from carbohydrates of which 7% were sugars, with a total MetE of 5237 kcal/kg, Supplementary Table S1) for 7 weeks to induce an obese phenotype. In the control group, virgin females received a standard diet (SD, Ssniff, Soest, Germany, catalog no. R/M-H V1534-0, composed of 9% MetE from fat, 24% MetE from protein, 67% MetE from carbohydrates of which 8.8% were sugars, with a total MetE of 3225 kcal/kg, Supplementary Table S1). Females were randomly assigned to each diet. Both groups were time-mated with males that received a SD and were kept on their respective chow during pregnancy and lactation. Litter size was randomly standardized to six pups. At the end of the lactation period on postnatal day 21 (P21)—a critical window of developmental programming—offspring were either sacrificed or weaned and kept on a SD until postnatal month 16 (P16M), when they were sacrificed. Animals were deeply anaesthetized using ketamine/xylazine and livers were harvested at each time point and weighed. The tissue was either fixed in 4% paraformaldehyde (PFA) or immediately stored at -80 °C until further processing. The exact numbers of animals are listed in the figure legends, a total of 58 offspring was investigated.

### Quantitative real-time reverse transcription polymerase chain reaction (qRT-PCR)

As previously described^24,51^, Tri-Reagent® (Sigma-Aldrich, Steinheim, Germany) was used to isolate ribonucleic acid (RNA) from frozen liver tissue following the manufacturer’s guidelines and RNA concentration was measured via the Tecan Spark (Tecan, Maennedorf,, Switzerland). Complementary deoxyribonucleic acid (cDNA) was generated and SYBR-Green or Taqman qRT-PCR was performed with the 7500 real-time PCR system (Applied Biosystems, Foster City, CA, USA) or the QuantStudio 5 (Applied Biosystems, Foster City, CA, USA). The utilized SYBR and Taqman primers are listed in Supplementary Table S2. ß-Actin served as reference gene and the 2 − ∆∆Ct method was performed to calculate the fold induction of mRNA expression compared to the control group.

### Western blot

For protein isolation, frozen samples were homogenized and extracted with a lysis buffer (CHAPS buffer, Millipore, MA, USA) as previously reported^24^. Protein concentration was quantified by a bicichoninic acid (BCA)-protein assay kit (Thermo Scientific, Waltham, MA, USA) and measured with the Tecan Spark (Tecan, Maennedorf, Switzerland). 10% Polyacrylamide gels were used for protein separation by gel electrophoresis (SDS-PAGE) and proteins were transferred to a nitrocellulose membrane with a semi-dry system. The applied primary antibodies for immunoblotting are listed in Supplementary Table S3. Anti-rabbit IgG, HRP-linked (Cell signaling, catalog no. 7074, Danvers, MA, USA), Anti-mouse IgG, HRP-linked (Cell Signaling, catalog no. 7076, Danvers, MA, USA), anti-rat IgG, HRP-linked (Cell Signaling, catalog no. 7077, Danvers, MA, USA) and anti-goat IgG, HRP-linked (Abcam, catalog no. ab6885, Cambridge, UK) were used as secondary antibodies. Quantitative analysis was performed via densitometry measurement using the Bio-Rad ImageLab software (Bio-Rad, Munich, Germany). Protein density was normalized by ß-actin or Glycerinaldehyd-3-phosphat Dehydrogenase (GAPDH).

### Histological analysis

PFA-fixed liver samples were embedded in paraffin. 3 µm thin sections were sliced and stained using hematoxylin and eosin (H&E) as previously described^52^. Images were taken with an Olympus BX43 microscope (Hamburg, Germany). The degree and type of steatosis, lobular inflammation and hepatocellular ballooning was evaluated by a liver pathologist blinded for the received diet and classified using the NAFLD-activity score (NAS)^53,54^.

### Immunofluorescence staining

PFA-fixed samples were sectioned into 3 µm thin slices and deparaffinized using Neo-Clear® (Sigma-Aldrich, St. Louis, MO, USA). Rehydration was performed using a descending ethanol series (100%, 96%, 80%, 70%) to PBS. The sections were treated with MaxBlock™ reagent A autofluorescence reducing kit (MaxVision Biosciences, DC, Kenmore, WA, USA) and washed with 60% ethanol and PBS afterwards. The samples were permeabilized using 1% Triton X100 in PBS for 15 min. Antigen Retrieval was performed using 1 mM EDTA (pH 8, Sigma-Aldrich, St. Louis, MO, USA) for 20 min. Blocking was performed with 3% peroxidase block for 10 min followed by SeaBlock blocking solution (Sea Block, Thermo Fisher Scientific, Waltham, MA, USA) for an hour. The primary antibody was applied overnight at 4 °C (rabbit anti-mouse γH2AX diluted 1:100 in antibody diluent (DAKO, Santa Clara, CA, USA), Supplementary Table S3). The sections were washed with PBS and incubated with a mouse anti-647 (Jackson Immuno Research, Cambridgeshire, United Kingdom, catalog no. 115-605-003, diluted 1:800) for an hour. The sections were treated with solution B from the MaxBlock autofluorescence reducing kit (MaxVision Biosciences, Washington, DC, USA) for 5 min at room temperature. DAPI dye (Sigma-Aldrich, St. Louis, MO, USA, catalog no. D9542, 1:5000) was used to counterstain the nucleus and sections were mounted with Fluoromount Aqueous mounting medium (Sigma Aldrich, St. Louis, MO, USA). Images were taken using an Olympus BX43 microscope (Hamburg, Germany) at 40X magnification (CellSens Dimension software; Olympus, Tokyo, Japan). The amount of γH2AX positive cells compared to all DAPI positive cells (%) of six fields of view per liver section was analyzed using Image J—Fiji (Version 1.54). The mean value was calculated.

### Proteomic analysis of the liver

For proteomics analysis, data from our group that was in part previously published was used^51^. Liver proteins were isolated as mentioned above. Protein acetone precipitation was performed, and samples were resuspended in 8 M urea buffer followed by reduction and alkylation of cysteines. Lysylendopeptidase (Lys-C) and trypsin were utilized for protein digestion. Styrene divinyl benzene- reversed-phase (SDB-RP) stage tips were used to purify the produced peptides. Samples were analyzed by the CECAD Proteomics Facility on an Orbitrap Exploris 480 (Thermo Scientific, granted by the German Research Foundation under INST 1856/71-1 FUGG) mass spectrometer equipped with a FAIMSpro differential ion mobility device that was coupled to an UltiMate 3000 (Thermo Scientific). Samples were loaded onto a precolumn (Acclaim 5 µm PepMap 300 µ Cartridge) for 2 min at 15 ul flow before reverse-flushed onto an in-house packed analytical column (30 cm length, 75 µm inner diameter, filled with 2.7 µm Poroshell EC120 C18, Agilent). Peptides were chromatographically separated at a constant flow rate of 300 nL/min and a linear gradient with initial 6% B (0.1% formic acid in 80% acetonitrile), up to 32% B in 72 min, up to 55% B within 7.0 min, followed by column wash with 95% solvent B and reequilibration to initial condition. The FAIMS pro was operated at -50 V compensation voltage and electrode temperatures of 99.5 °C for the inner and 85 °C for the outer electrode. Sample data were acquired using MS1 scans were from 390 to 1010 m/z at 15 k resolution. Maximum injection time was set to 22 ms and the AGC target to 100%. MS2 scans ranged from 400 to 1000 m/z and were acquired at 15 k resolution with a maximum injection time of 22 ms and an AGC target of 100%. DIA scans covering the precursor range from 400 to 1000 m/z and were acquired in staggered 75 × 8 m/z windows, resulting in nominal 4 m/z windows after deconvolution using ProteoWizard^55^. All scans were stored as centroid. For the gas-phase fractionated library^56^, a pool generated from all samples was analyzed in six individual runs covering the range from 400 to 1000 m/z in 100 m/z increments. For each run, MS1 was acquired at 60 k resolution with a maximum injection time of 98 ms and an AGC target of 100%. MS2 spectra were acquired at 30 k resolution with a maximum injection time of 60 ms. Spectra were acquired in staggered 4 m/z windows, resulting in nominal 2 m/z windows after deconvolution. The gas-phase fractionated library was build using DIA-NN 1.8^57^ using a Swissprot mouse canonical database (UP589, downloaded 04/01/21) with settings matching acquisition parameters. Samples were analyzed in DIA-NN 1.8 as well using the previously generated library and identical database. DIA-NN was run with the additional command line prompts “—report-lib-info” and “—relaxed-prot-inf”. Further output settings were: filtered at 0.01 FDR, N-terminal methionine excision enabled, maximum number of missed cleavages set to 1, min peptide length set to 7, max peptide length set to 30, min precursor m/z set to 400, max precursor m/z set to 1000, cysteine carbamidomethylation enabled as a fixed modification. Afterwards, DIA-NN output was further filtered on library q-value and global *q*-value ≤ 0.01 and at least two unique peptides per protein using R (4.1.3). Finally, LFQ values calculated using the DIA-NN R-package. Downstream analysis was performed using the R version 2023.09.1 + 494. Only proteins that were identified in at least all replicates of one condition were included in the analysis. The ‘DEP’ package was used to normalize the data and impute missing values using the MinProb approach before performing a differential enrichment analysis^58^. Gene-set enrichment analysis (GSEA) was conducted based in the assigned gene names for each identified protein. The ranking was performed using the sign of the mean log_2_FC between SD and HFD and the log_10_ p value . All hallmark gene sets and Gene Ontology (GO) gene sets were used for GSEA. For over-representation analysis (ORA), significantly upregulated proteins in HFD-samples were included and an ORA was performed based on the GO gene sets. Both analyses were performed using ‘clusterProfiler’, ‘msigdbr’, ‘org.Mm.eg.db’ and ‘biomaRt’^59,60^. The results were visualized using ‘enrichplot’ and ‘ggplot2’ packages.

### Statistical analysis

The data is presented as mean ± standard error of mean (SEM). Statistical analysis of histological analysis, immunofluorescence staining, qRT-PCR and Western Blot was performed using Graph Pad Prism (GraphPad version 10.0, San Diego, CA, USA). Normal distribution of the data was tested using the D’Agostino & Pearson test, or for smaller datasets, the Shapiro–Wilk test. T-test was conducted for parametric, and Mann–Whitney-U-test was performed for non-parametric data. Categorial variables were analyzed by Fisher’s exact test and chi-square test. The statistical significance level (*p* < 0.05) was indicated as *p < 0.05, **p < 0.01, and ***p < 0.001. Figures were created with Adobe Illustrator.

## Supplementary Information

Below is the link to the electronic supplementary material.

Supplementary Information.

## Data Availability

The data are not publicly available due to ethical restrictions and legal constraints. Readers may contact the corresponding authors for reasonable requests for the data. De-identified data may be provided after approval from the ethical review board.
