# Supplementary material for: Perinatal obesity primes the hepatic metabolic stress response in the offspring across life span
Source: Sci Rep. 2025 Feb 21;15:6416. doi: 10.1038/s41598-025-90082-4 (PMC11845730; doi:10.1038/s41598-025-90082-4)
Supplement: Supplementary file 1 — Supplementary Information. [file 41598_2025_90082_MOESM1_ESM.pdf]

Perinatal obesity primes the hepatic metabolic stress response in the offspring across life span

Sarah K Stegmann, Christina Vohlen, Nam Gyu Im, Jana Niehues, Jaco Selle, Ruth Janoschek, Celien Kuiper-Makris, Sonja Lang, Münevver Demir, Hans-Michael Steffen, Alexander Quaas, Jan-Wilm Lackmann, Dirk Nierhoff, Christoph Neumann-Haefelin, Jörg Dötsch, Miguel Alejandre Alcazar, Philipp Kasper

Supplementary Files

Late adulthood

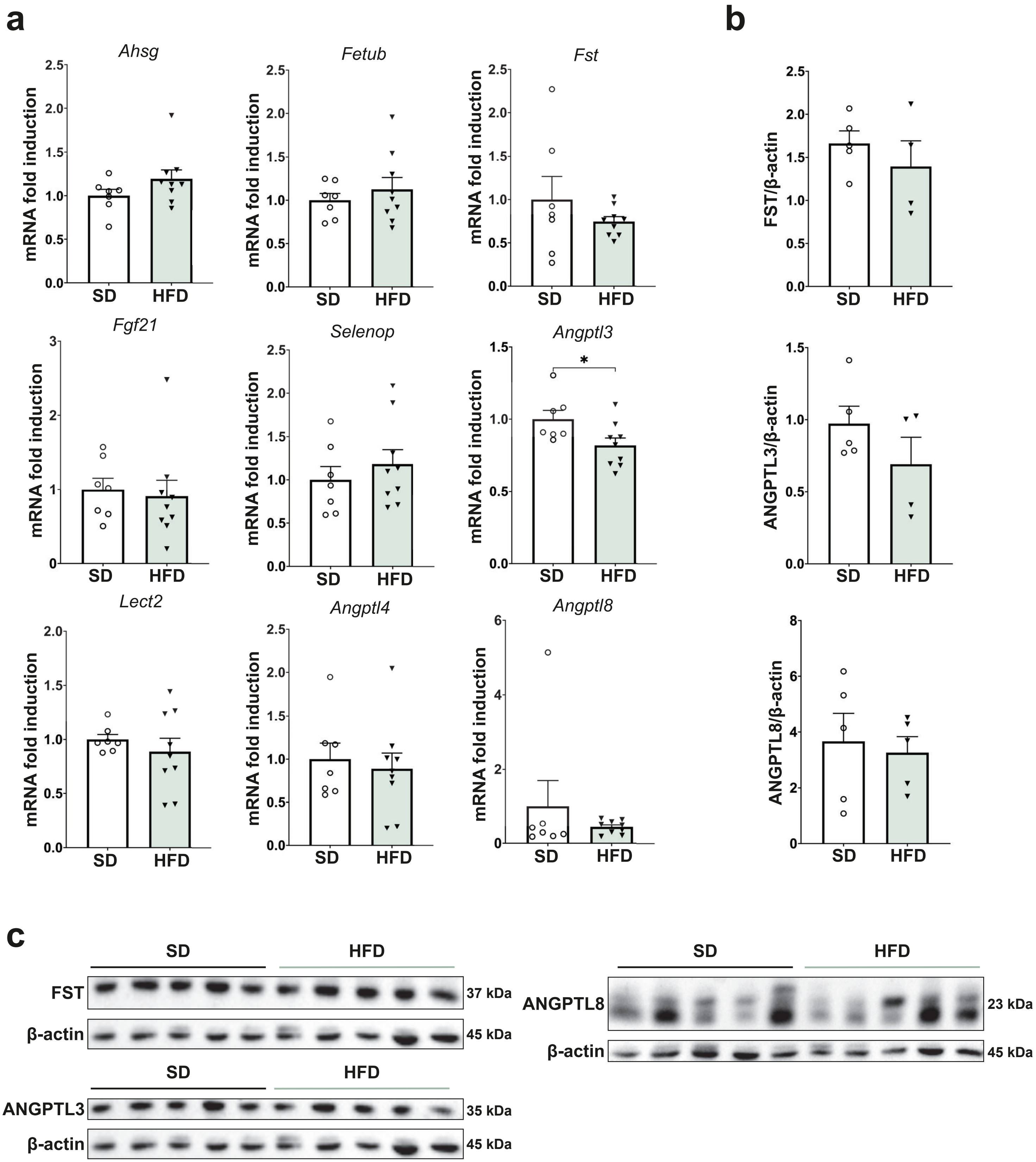

Supplementary Figure S1. Hepatokine profile of offspring of perinatal obesity in late adulthood
a) The hepatokine profile of offspring of perinatal obesity is mostly restored in late adulthood on mRNA level while ANGPTL3 is still downregulated (SD n=7, HFD n=9). b-c) Western Blot analysis of follistatin, ANGPTL3 and ANGPTL8 in late adulthood. Uncropped images of original blots are provided in Supplementary Figure S4. Data are presented as mean ± SEM. T-Test and Mann-Whitney U-Test was performed and statistical significance was defined as $p<0.05$.

Female offspring - early life

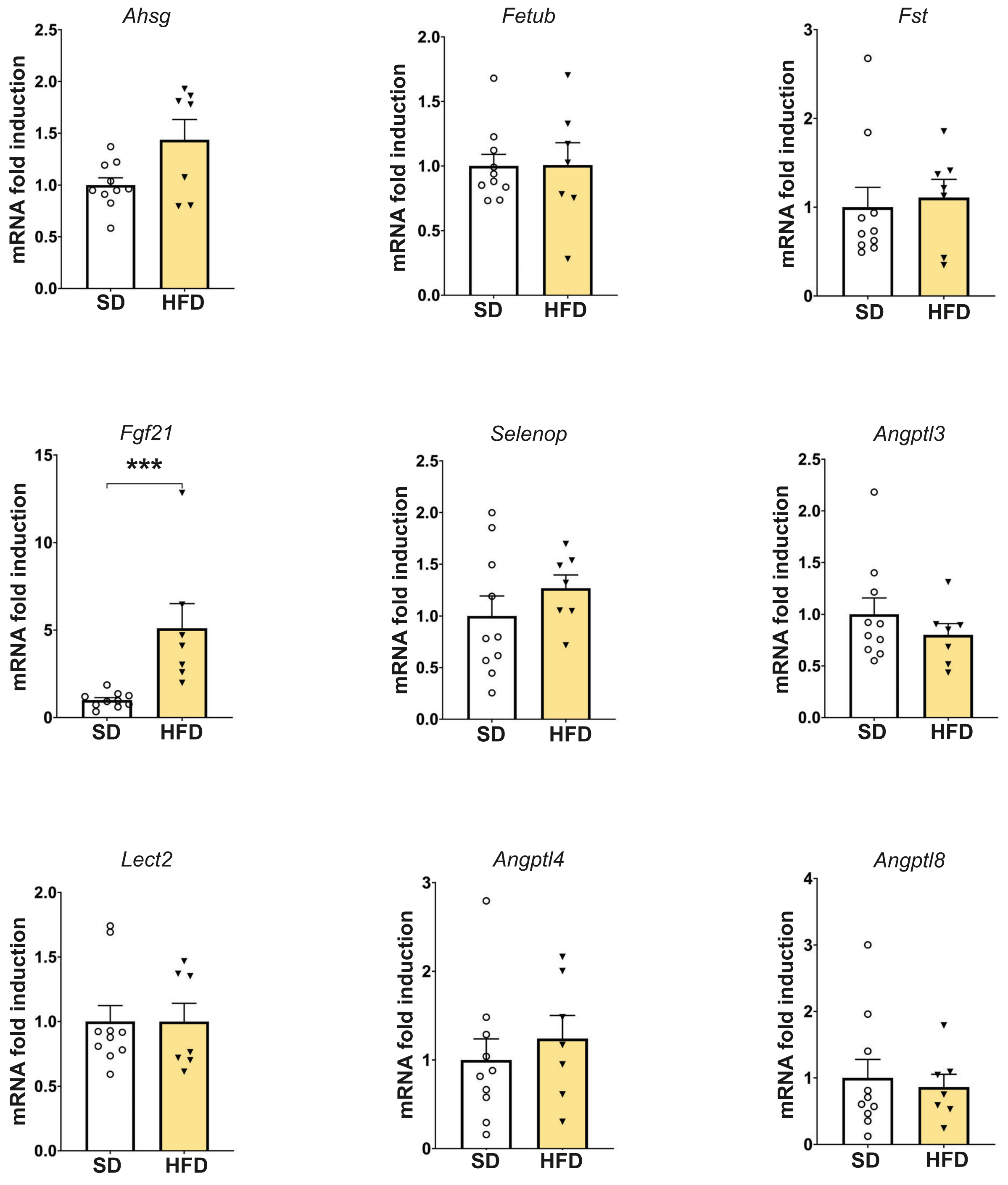

Supplementary Figure S2. Hepatokine profile of female offspring of perinatal obesity in early life
The hepatokine profile of offspring of perinatal obesity in early life on mRNA level (SD n=10, HFD n=7). Data are presented as mean ± SEM. T-Test and Mann-Whitney U-Test was performed and statistical significance was defined as $p<0.05$.

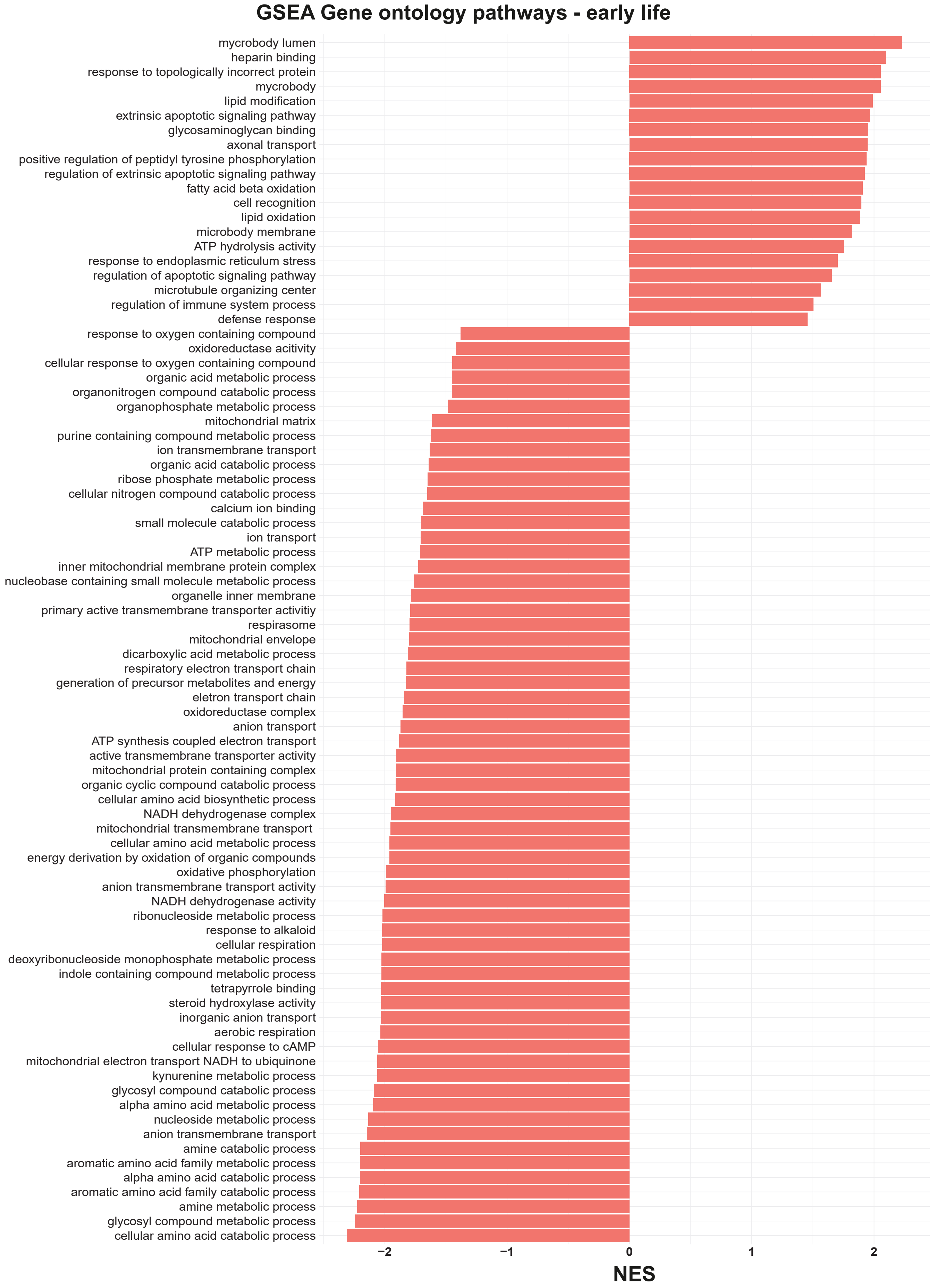

Supplementary Figure S3. Gene set enrichment analysis (GSEA) of Gene ontology (GO) gene sets reveals an altered lipid metabolism after perinatal obesity

GSEA of proteomics data obtained in early life using the GO gene sets (n=5 per group). All significantly altered pathways are displayed with their normalized enrichment score (NES). 'Lipid modification', 'fatty acid beta oxidation' and 'lipid oxidation' pathways are significantly enriched in offspring of perinatal obesity. GSEA was performed and statistical significance was considered as p.adjust<0.05

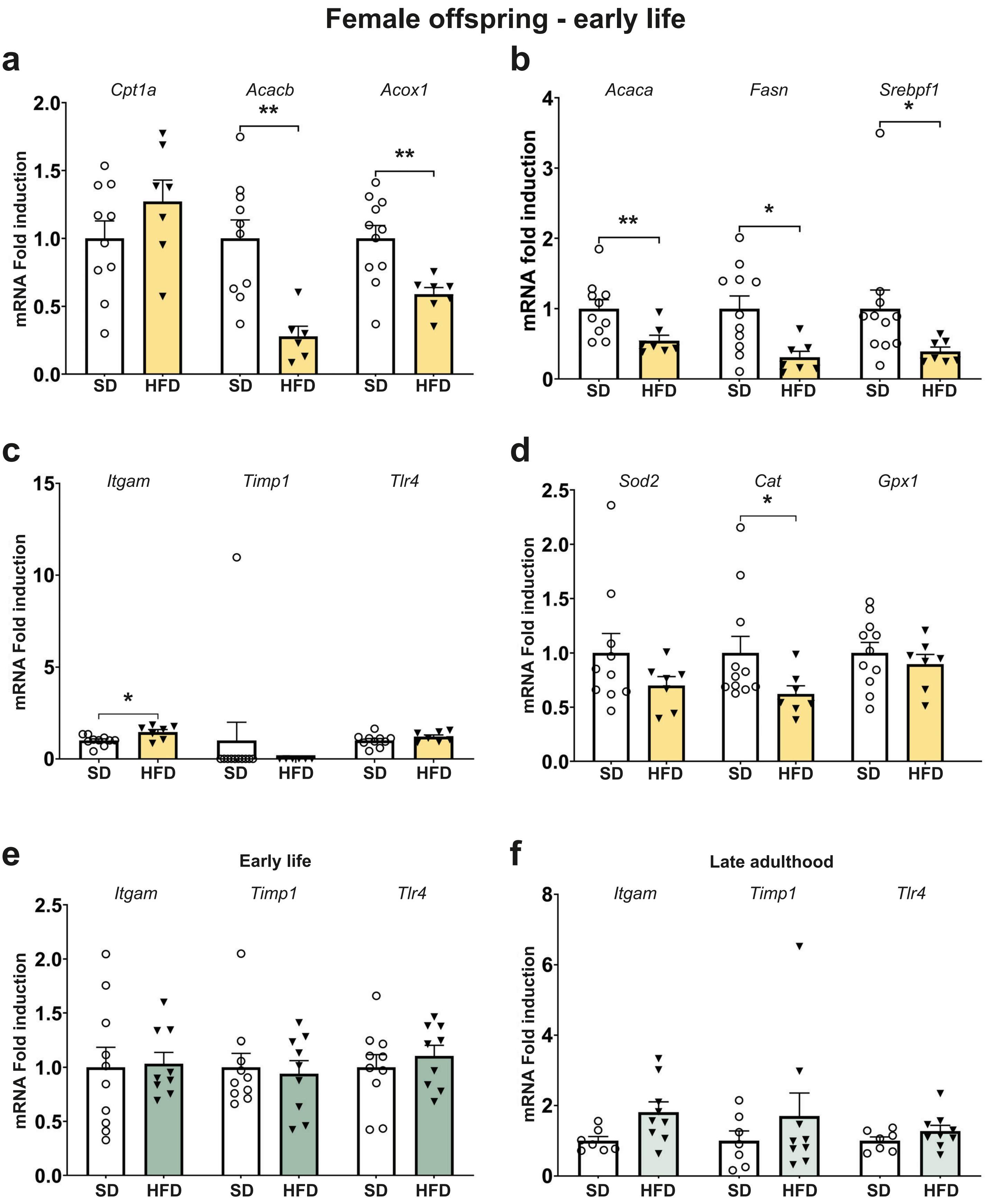

Supplementary Figure S4. Perinatal HFD leads to an altered hepatic stress response in female offspring in early life

a) Quantitative real-time PCR of female offspring show an altered fatty acid oxidation in early life after perinatal HFD. b) Key regulatory enzymes of lipogenesis are downregulated in early life. c) Oxidative stress markers in female offspring indicate an altered oxidative stress level. d) Antioxidative enzymes are decreased (SD n=10, HFD n=7). e-f) Quantitative real-time PCR performed in early life (SD n=10, HFD n=9) and late adulthood (SD n=7, HFD n=9) of markers for oxidative stress are not differently expressed. Data are presented as mean ± SEM. T-Test and Mann-Whitney U-Test was performed and statistical significance was defined as $p<0.05$, marked as *.

Uncropped images of western blots

Figure 2b

FST

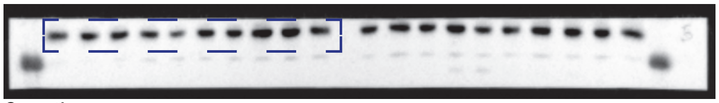

β-actin

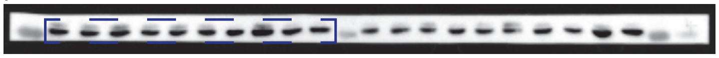

ANGPTL8

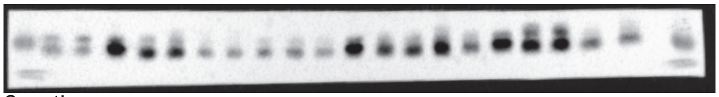

β-actin

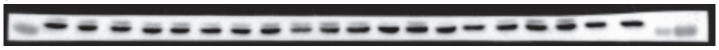

ANGPTL3

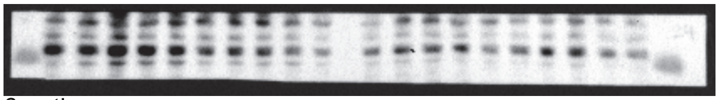

β-actin

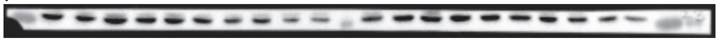

Figure 4c

pACC

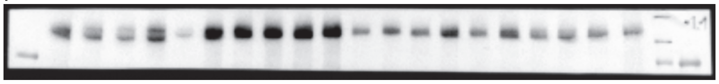

ACC

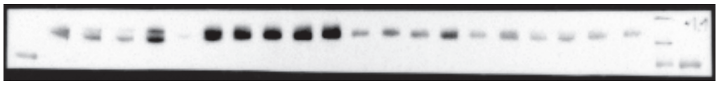

FAS

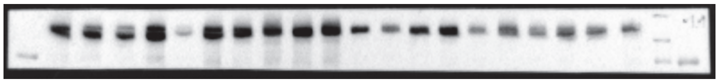

SREBP1

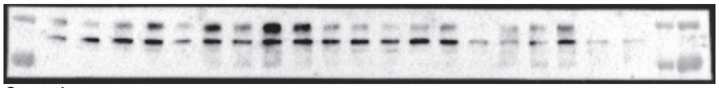

β-actin

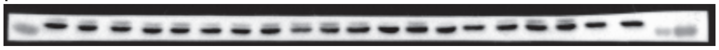

Figure 4e

pACC

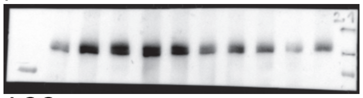

ACC

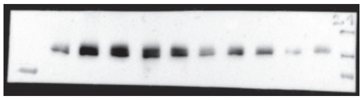

FAS

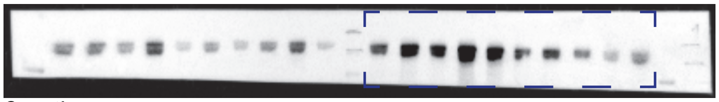

β-actin

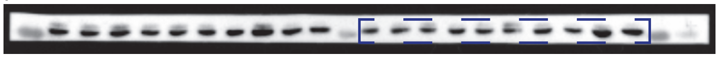

SREBP1

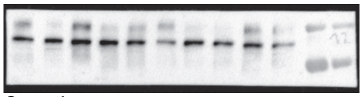

β-actin

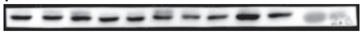

Figure 5a

4-HNE

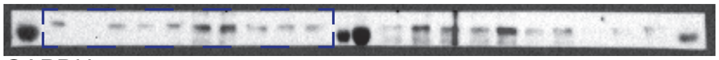

GAPDH

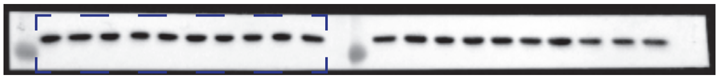

Figure 5b

4-HNE

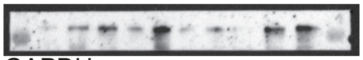

GAPDH

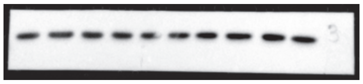

Figure 5c-d

MDA

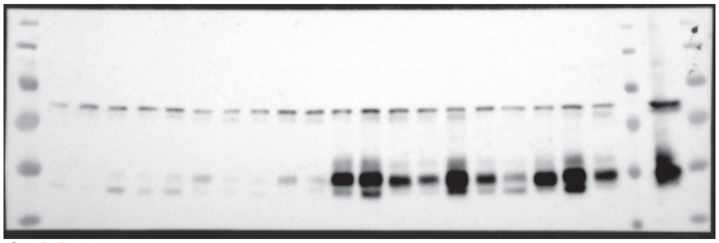

GAPDH

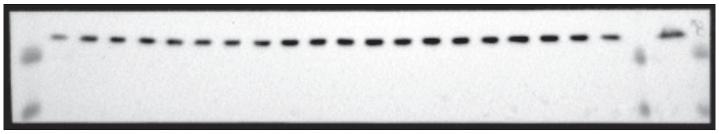

Figure 5f

Oxphos early life

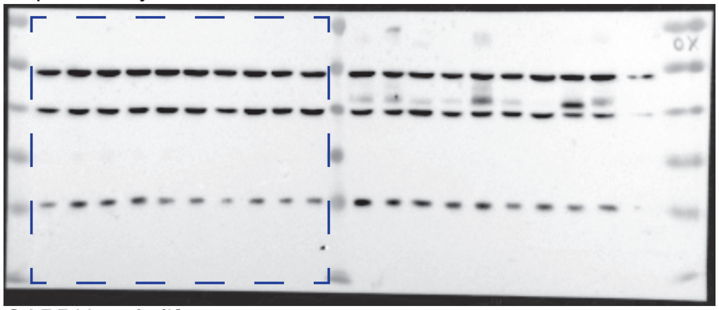

GAPDH early life

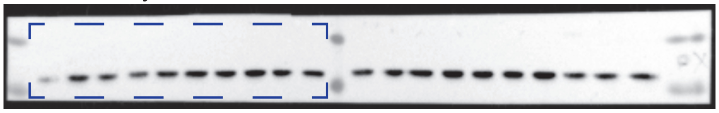

Oxphos late adulthood

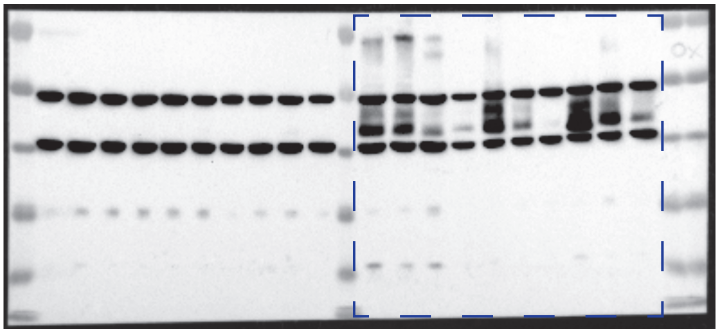

GAPDH late adulthood

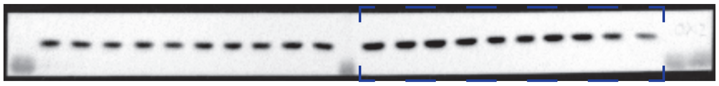

Figure 6d-e

CAT

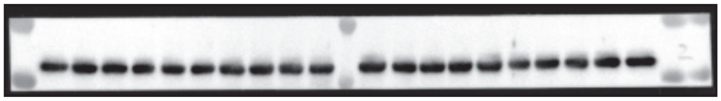

SOD2

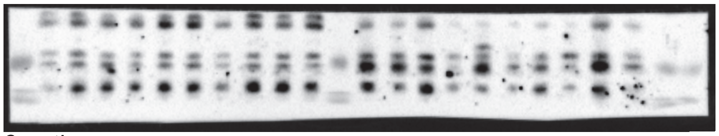

β-actin

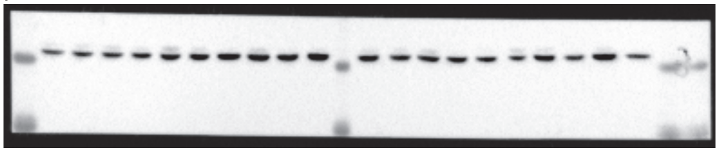

Supplementary Figure S1

FST

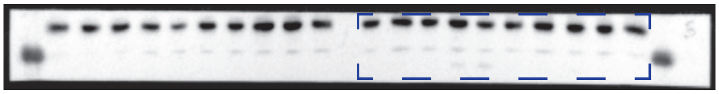

ANGPTL3

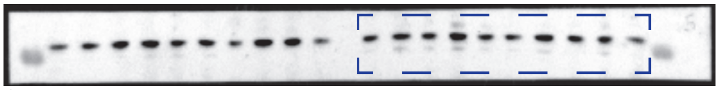

β-actin

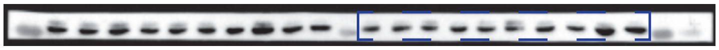

ANGPTL8

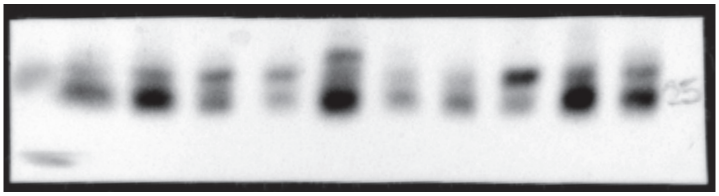

β-actin

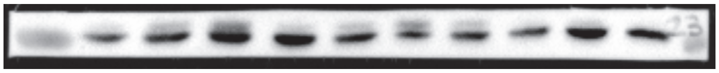

Supplementary Figure S5. Uncropped images of original blots displayed in the main figures

Supplementary Table S1. Diet composition of SD and HFD

	SD	HFD
Company	Ssniff: V1534 – R/M-Maintenance	Altromin: C1057, modified
Metab. energy (kcal/kg)	3225	5237
Metab. energy (MJ/kg)	12.8	21.9
Fat (kJ%)	9.0	60.0
Protein (kJ%)	24.0	16.0
Carbohydrates (kJ%)	67	24.0
Sugar (sucrose) (kJ%)	8.8	7.0
Crude ingredients (g/100g)		
Fat (g/100g)	3.3	35.1
Protein (g/100g)	19.0	20.8
Carbohydrates (g/100g)	41.3	41.7
Sugar (g/100g)	5.3	12.1

Supplementary Table S2. Primer sequences used for qRT-PCR

Gene	Primer	Sequence
Acaca	for	GAAGTCAGAGCCACGGCACA
	rev	GGCAATCTCAGTTCAAGCCAGTC
Acacb	for	CTACAAGACGGCGCAGGTCA
	rev	AGGCGCCAAACTTCAGCATC
Acox1	for	GAGCCTTTGGACCTTCACTTGG
	rev	CGCATAAGTGCCCGTGATCT
Angptl3	for	ACGAAAAGGGCTTTGGGAGG
	rev	TCAACGTAGTGCTTGCTGTCT
Angptl4	for	CAGCTCATTGGCTTGACTCC
	rev	AGGCTGGATCTGGAAAAGTCC
Angptl8	for	CGGGACACTGTACGGAGACTA
	rev	GTGAGAGCCCATAAGAGGTGG
Cat	for	CCATCCTTTATCCATAGCCAGAA
	rev	GAATCCCTCGGTCACTGAACAA
Cpt1a	for	CATTACAAGGACATGGGCAAGTT
	rev	CCGTAGTGCAGGAGCGTACA
Fasn	for	CCATGGAGCGTATATGTGAACAG
	rev	AATGCCCACGTCACCAATG
Ahsg	for	TCAGATCGACAAAGTCAAGGTGT
	rev	GTCAGCTGCCTCACAGAACAGT
Fetub	for	GGCTATGAACCAGTGGGTGT
	rev	TTGGACCGTAGAACCTTGGC
Fgf21	for	CAGGGAGGATGGAACAGTGGTA
	rev	GCTGTTGGCAAAGAAACCTAGAG
	taq	AGCACACCGCAGTCCAGAAAGTCTCC
Follistatin	for	CTGAGAAAGGCCACCTGCTT
	rev	CACACTGGATATCTTCACAGGACT
Gpx1	for	GACACCAGGAGAATGGCAAGA
	rev	TTCTCACCATTCACTTCGCACTT
Itgam	for	AGTGCTGGGAGACGTGAATG
	rev	GCACTGAGGCTGGCTATTGA
Lect2	for	ATGGCATTCGACTGTCTGGAA
	rev	GGTAAACTTTCTGCAGGGGC
Selenop	for	CAGGACGAAGCTAGTCCGAAG
	rev	GGGCTTTGTAACAAGCAGAGC
Sod2	for	GCCTGCTCTAATCAGGACCC
	rev	AGACTACAGCACCCCAGTCA
Srebpf1	for	CATCGACsTACATCCGCTTCTTG
	rev	GTGATTTGCTTTTGTGTGCACTTC
Timp1	for	ACCTGGTCATAAGGGCTAAATTCA
	rev	CCGGATATCTGCGGCATT
Tlr4	for	GGTGAGAAATGAGCTGGTAAAGAATT
	rev	GCAATGGCTACACCAGGAATAAA

Supplementary Table S3. Primary antibodies used for western blot analysis

Protein	Concentration	Company
4-HNE	1:1000	R&D Systems, Minneapolis, MN, USA catalog no. MAB3249
ACC	1:2000	Cell signaling, Danvers, MA, USA catalog no. 3662
β-actin	1:10,000	Cell signaling, Danvers, MA, USA catalog no. 3700
ANGPTL3	1.25:1000	Novus Biologicals, Centennial, CO, USA catalog no. AF136
ANGPTL8	1:500	Novus Biologicals, Centennial, CO, USA catalog no. MAB8548
Catalase	1:1000	Abcam, Cambridge, UK catalog no. ab1877
FAS	1:1000	Cell signaling, Danvers, MA, USA catalog no. 3180
Follistatin	1:1000	Abcam, Cambridge, UK catalog no. ab64490
γH2AX (Phospho S139)	1:200	BioLegend, San Diego, CA, USA catalog no. 613402
GAPDH	1:3000	Cell signaling, Danvers, MA, USA catalog no. 2118
MDA	1:1000	Abcam, Cambridge, UK catalog no. ab243066
Oxphos	1:2000	Abcam, Cambridge, UK catalog no. ab110413
pACC	1:1000	Cell signaling, Danvers, MA, USA catalog no. 3661
SOD2	1:1000	Abcam, Cambridge, UK catalog no. 3108
SREBP1	1:1000	Thermo Fisher Scientific, Waltham, MA, USA catalog no. MA5-16124
